# Characterization of the RNA Mycovirome Associated with Grapevine Fungal Pathogens: Analysis of Mycovirus Distribution and Their Genetic Variability within a Collection of *Botryosphaeriaceae* Isolates

**DOI:** 10.3390/v16030392

**Published:** 2024-03-01

**Authors:** Gwenaëlle Comont, Chantal Faure, Thierry Candresse, Marie Laurens, Sophie Valière, Jérôme Lluch, Marie Lefebvre, Sébastien Gambier, Jérôme Jolivet, Marie-France Corio-Costet, Armelle Marais

**Affiliations:** 1 UMR Santé et Agroécologie du Vignoble (1065), ISVV, Labex Cote, Plant Health Department, INRAE, 33140 Villenave d’Ornon, France; gwenaelle.comont@inrae.fr (G.C.); marie.laurens@inrae.fr (M.L.); sebastien.gambier@inrae.fr (S.G.); jerome.jolivet@inrae.fr (J.J.); 2 UMR BFP, INRAE, University of Bordeaux, 33140 Villenave d’Ornon, France; chantal.faure@inrae.fr (C.F.); thierry.candresse@inrae.fr (T.C.); marie.lefebvre@inrae.fr (M.L.); 3 INRAE, US 1426, GeT-PlaGe, GenoToul, 31320 Castanet-Tolosan, France; sophie.valiere@inrae.fr (S.V.); jerome.lluch@inrae.fr (J.L.)

**Keywords:** grapevine trunk disease, *Diplodia*, *Neofusicoccum*, *Lasiodiplodia*, *Botryosphaeria*, mycovirus, high-throughput sequencing

## Abstract

*Botryosphaeriaceae* are fungi involved in the decay of various woody species, including the grapevine, leading to significant production losses. This fungal family is largely ubiquitous, and seven species of *Botryosphaeriaceae* have been identified in French vineyards, with variable levels of aggressiveness, both in vitro and in planta. Mycoviruses can impact the life traits of their fungal hosts, including aggressiveness, and are one of the factors influencing fungal pathogenicity. In this study, the RNA mycovirome of fifteen *Botryosphaeriaceae* isolates was characterized through the high-throughput sequencing of double-stranded RNA preparations from the respective samples. Eight mycoviruses were detected, including three potential novel species in the *Narnaviridae* family, as well as in the proposed Mycobunyaviridae and Fusagraviridae families. A large collection of *Botryosphaeriaceae* isolates was screened using RT-PCR assays specific for 20 *Botryosphaeriaceae*-infecting mycoviruses. Among the mycoviruses detected, some appeared to be specialists within a single host species, while others infected isolates belonging to multiple *Botryosphaeriaceae* species. This screening allowed us to conclude that one-third of the *Botryosphaeriaceae* isolates were infected by at least one mycovirus, and a significant proportion of isolates (43.5%) were found to be coinfected by several viruses, with very complex RNA mycoviromes for some *N. parvum* isolates.

## 1. Introduction

*Botryosphaeriaceae* is a family of ascomycetous fungi (*Dothiomycetes*) in the order *Botryosphaeriales*, comprising numerous genera and species [1] that are widely distributed throughout the world. These fungi are pathogens of numerous perennial plant species (fruit trees and forest trees), including the grapevine, and are generally endophytic [2]. Due to their opportunistic and ubiquitous nature, they generally have a very wide host range and are sometimes considered asymptomatic latent pathogens. Their pathogenicity is essentially expressed after the abiotic stress of the host plant, which favors their development and can lead to the death of the host [3]. As a result, in the context of global changes in agriculture, these fungi could become a major disease challenge to be addressed.

The grapevine, *Vitis vinifera*, is affected by these pathogens, alone or in association with other fungi, resulting in grapevine trunk diseases (GTDs) [4], which are considered highly damaging to the world’s vineyards, with an estimated economic cost of over USD 1.5 billion per year. These pathogens can lead to vine death, thereby reducing vineyard longevity, impacting yields, and causing heterogeneity associated with replanting. The most common symptoms due to *Botryosphaeriaceae* are central or sectorial necrosis in the wood, a brown stripe on the trunk, the presence of cankers, and very sudden leaf discoloration with desiccation [5]. 

More than 22 species of *Botryosphaeriaceae* have been recorded on grapevine, with different distributions depending on the country and climate [6,7,8]. Among the *Botryosphaeriaceae* genera and species described in vineyards, *Diplodia seriata* is the most abundant species, along with *Neofusicoccum parvum*, *Lasiodiplodia theobromae*, and *Botryosphaeria dothidea* [4,9]. More recently, *Lasiodiplodia viticola*, *Spencermartinsia viticola*, and *Diplodia intermedia* have been described as present in vinewood collected from French vineyards [10]. The biology of these fungi is still poorly understood [11], but the toxins produced are thought to be responsible for foliar symptoms, and depending on the strain or species, the toxins produced may differ and play a role in pathogenicity [12,13]. 

To date, there has been no truly effective solution for the control of *Botryosphaeriaceae* in vineyards since the ban on sodium arsenite in 2001 [4,14], and the incidence of GTDs has increased in France over the last few decades but to varying degrees depending on the region and grape variety [15]. Many research projects are currently underway to develop control methods or combinations thereof, including the search for potential biocontrol agents or products (e.g., *Trichoderma*, *Bacillus*, *Pythium*, chitosan, and polyphenols) [4,16]. However, *Botryosphaeriaceae*, as ubiquitous fungi not limited to a single host species, are able to degrade many molecules, including polyphenols, or they are not very sensitive to them [17,18]. One possibility would be to explore the potential of the mycoviruses present in these species and assess their potential use as biocontrol agents, following the approach applied to *Cryphonectria parasitica*, responsible for chestnut canker [19]. 

Over the past 15 years, the interest in mycovirus research has been intensely reignited mainly due to the advent of high-throughput sequencing (HTS) technologies, enabling the efficient screening of mycoviruses from mycelia without any a priori knowledge. This renewed interest also stems from the potential of mycoviruses as biocontrol agents of plant pathogenic fungi, even though it is generally accepted that most mycoviruses cause latent infections without any clear effect on their fungal hosts [20,21]. HTS studies have shown mycoviruses to be widespread within the fungal kingdom. According to recent taxonomic advances, there are currently more than 250 recognized mycovirus species [22,23], and this number is rapidly growing. Most mycoviruses are positive-sense, single-stranded RNA (+ssRNA) or double-stranded RNA (dsRNA) viruses, although a few negative-sense single-stranded RNA (-ssRNA) and single-stranded DNA (ssDNA) viruses have also been described (for review, see [24]). Regarding fungal hosts, most mycoviruses have been identified from the *Sclerotiniaceae* family [22]. Considering the grapevine-associated *Botryosphaeriaceae* species, mycovirus screening has been mainly conducted in *B. dothidea*, probably because this fungus represents one of the most economically important phytopathogenic fungi worldwide, with a broad host range, including fruit trees and grapevine [2]. So far, 18 mycoviruses have been described from various strains of *B. dothidea:* Ten of them are dsRNA viruses from four families (*Chrysoviridae*, *Partitiviridae*, *Totiviridae*, and the proposed Botybirnaviridae), and two are unassigned [25,26,27,28,29,30,31,32,33]. The remaining eight viruses are +ssRNA viruses in the families *Alphaflexiviridae* [34], *Fusariviridae* [35,36], *Mitoviridae* [37,38,39] and *Botourmiaviridae* [40,41,42]. Some of these mycoviruses have been identified from hypovirulent strains of *B. dothidea*, suggesting that they might have a negative impact on the fungal host virulence [25,28,32,34]. Very interestingly, a set of novel viroid-like RNAs called “mycoviroids” have also been discovered from *B. dothidea,* and it has been suggested that they could modulate some biological traits of the fungal host such as virulence and growth rate [43]. In *N. parvum*, several mycoviruses have also been characterized: three species each in the *Mitoviridae, Totiviridae*, and *Narnaviridae* families; one species each in families *Botourmiaviridae*, *Endornaviridae*, and *Chrysoviridae*; and one unclassified +ssRNA virus [44,45,46]. However, these studies have not provided any clues regarding the potential impact of *N. parvum*-infecting mycoviruses on their host biology. The recent work of Khan et al. [47], aiming to characterize the virome of a single loquat isolate of *D. seriata*, allowed for the characterization of eight viruses belonging to seven families (*Polymycoviridae*, *Chrysoviridae*, *Totiviridae*, *Partitiviridae*, *Botourmiaviridae*, and the proposed Ambiguiviridae and Spilpalmiviridae families), three of them potentially affecting fungal colony phenotype. In addition, an *Endornaviridae* member was previously described from a *D. seriata* isolate from an asymptomatic grapevine plant [44]. 

In the present work, using an HTS-based approach and classical RT-PCR assays, we investigated the RNA mycovirome of *Botryosphaeriaceae* isolates from a collection of 69 isolates representing 13 species. Most of the isolates were collected from asymptomatic or symptomatic wood disease grapevine plants in French vineyards, or they were obtained from the Westerdijk Institute as CBS isolates (CBS for Centraal Bureau voor Schimmelcultures). The distribution of 20 *Botryosphaeriaceae*-infecting mycoviruses and their genetic diversity in a collection of over 60 fungal isolates were also assessed.

## 2. Materials and Methods

### 2.1. Growth Conditions and Characterization of Botryosphaeriaceae Isolates

The majority of the isolates (*n* = 43) came from the national grapevine trunk disease survey carried out in French vineyards between 2003 and 2008 [15] and constitute the main part of the “CoCo” collection isolated and processed in the SAVE laboratory [10]. In addition, the isolates collected before this date (*n* = 9) and CBS isolates (*n* = 16) from the Westerdijk Institute were also analyzed (Table 1). In total, 69 isolates belonging to 5 genera and 13 *Botryosphaeriaceae* species [*Botryosphaeria dothidea* (*n* = 3), *Diplodia intermedia* (*n* = 2), *Diplodia mutila* (*n* = 10), *Diplodia rosulata* (*n* = 1)*, Diplodia sapinea* (*n* = 1), *Diplodia scrobiculata* (*n* = 1), *Diplodia seriata* (*n* = 23), *Lasiodiplodia pseudotheobromae* (*n* = 2), *Lasiodiplodia viticola* (*n* = 3), *Neofusicoccum luteum* (*n* = 2), *Neofusicoccum parvum* (*n* = 15), *Neofusicoccum ribis* (*n* = 2), and *Spencermartinsia viticola* (*n* = 4)] were included in this study (Table 1). All the isolates from French vineyards (different regions and cultivars) were sampled from either symptomatic or asymptomatic plants, and cultivated from bark, necrosis tissue, or from healthy parts proximal to necrosis (Table 1). All isolates were routinely stored at 5 °C on a malt agar (MA) medium as described previously [10]. 

Following morphological characterization, *Botryosphaeriaceae* isolates were molecularly identified at the species level by amplifying and sequencing the 5′ end of the large ribosomal subunit gene using the primers NL1-NL4 [48] and the internal transcribed spacer region using the universal primers ITS1-ITS4 [49]. In addition, in order to complete the identification of some isolates, a portion of the β-tubulin gene was also analyzed using the primers Bt2a-Bt2b [50] and a part of Ef1-α gene using the primers EF1-728F-EF1-986R [51]. At least 100 mg of dry-weight mycelium was collected and used for nucleic acid extraction as previously described [45].

### 2.2. Determination of Fungal Isolates’ In Vitro Growth Rates

From mycelium grown on the MA medium at 22 °C, a mycelial plug was deposited on a new Petri dish and incubated at 22 °C for three days. Then, the method described by Bellée et al. [13] was followed. Briefly, mycelial plugs (5 mm diameter) were transferred to the MA medium and incubated under controlled conditions at 28 °C with 16/8 h day/night photoperiod in a growth cabinet (LMS^TM^, Fisher Scientific, Illkirch, France). The radial growth was measured on a daily basis (two perpendicular diameters of the mycelium) and used to calculate the AUCs (areas under the curve) for each fungal isolate and each growth temperature. The AUCs were calculated using the following formula AUC = Σ (X_i_ + X_i+1_)/2(t_i+1_ − ti) [52], where t is the time of each reading, and Xi is the radial growth (mm) at time i. The experiment was carried out in triplicate. The averages of growth measurement for each isolate at each temperature were subjected to statistical analyses using a nonparametric test (Kruskal–Wallis) and significant differences were determined with a paired-sample Wilcoxon test at the 5% significance level using the R 3.0.3 software.

### 2.3. Pathogenicity Assays: Length of Necrosis

The cuttings of *V. vinifera* cv. Cabernet Sauvignon were rooted and potted in a greenhouse and two-month-old plants, and 10–12 leaves were used for the experiments [13]. After stem perforation, each plant was inoculated by depositing an MA plug with or without mycelium. Fifteen to twenty plant cuttings were inoculated per isolate and placed in the greenhouse with a 16 h/8 h day/night photoperiod and drip watering. Four months after inoculation, stems were longitudinally cut to measure the internal necrosis length (lesion inside the wood tissue) [13]. A re-isolation test (five repetitions) was performed from the internal necrotic zones of the inoculated plants, as previously described [13].

### 2.4. Double-Stranded RNA Extraction and High-Throughput Sequencing Analysis

Lyophilized fungal mycelia were powdered in the presence of liquid nitrogen and sterile sand in a precooled mortar. Double-stranded RNAs were then purified according to the protocol described in Marais et al. [53], before being analyzed by Illumina sequencing in a multiplexed format as previously described [54]. 

After demultiplexing and quality trimming, the reads were de novo-assembled into contigs using the CLC Genomics Workbench (CLC-GW, Qiagen, Courtaboeuf, France) and the following assembly parameters: word size: 50, bubble size: 300, and minimal contig length: 250. Contigs were then annotated via BlastN and BlastX analysis against nonredundant GenBank databases. Alternatively, the cleaned reads were mapped on viral reference sequences (RefSeq https://www.ncbi.nlm.nih.gov/refseq/ (accessed on 18 May 2023)) or on the identified viral contigs using CLC-GW and stringent parameters (in general >90% of reads length with >90% nucleotide identity). 

### 2.5. Completion of Genomic Sequences

When needed, identified contigs were extended by rounds of mapping of residual reads in CLC-GW. Genome ends (5′ and 3′) were determined using the rapid amplification of cDNA ends (RACE) strategy and the internal primers designed from the corresponding contigs, following the manufacturer’s instructions (Takara Bio Europe/Clontech^©^, Saint-Germain-en-Laye, France). PCR products were then directly Sanger-sequenced, and the sequences were finally assembled with the initial contigs to generate the complete viral genomic sequences.

### 2.6. Sequence and Phylogenetic Analyses

Phylogenetic and molecular analyses were conducted using MEGA version 11.0 [55]. Maximum likelihood trees were reconstructed from MUSCLE alignments using FastTree [56]) and the LG model [57], and randomized bootstrapping was performed for the evaluation of the validity of branches. 

### 2.7. Total Nucleic Acid (TNA) Extraction and the Detection of Mycoviruses by RT-PCR

TNAs were extracted from 3-day-old fungal cultures grown at 22 °C on a cellophane film (Hutchinson, Chalette/Loing, France) overlaid on MA plates as previously described [45]. The detection of 20 *Botryosphaeriaceae*-infecting mycoviruses was accomplished through two-step RT-PCR using the specific primers designed from the HTS sequences for the viruses characterized in this study or from the sequences of the grapevine *Botryosphaeriaceae*-infecting viruses described in previous studies [44,45,54,58] (Table S1). TNAs were reverse-transcribed into cDNA using a mixture of dT_18_ and N_6_ as reverse primers and the reverse transcriptase RevertAid H minus (Thermo Scientific, Illkirch, France) according to Marais et al. [59]. The cDNA was then submitted to PCR amplification using specific primers targeting individual mycoviruses (Table S1). PCR products were visualized on a 1.5% agarose gel, and their nucleotide sequence was determined via the direct Sanger sequencing of amplicons (Eurofins Genomics, Ebersberg, Germany).

## 3. Results

### 3.1. RNA Virome Associated to Botryosphaeriaceae Species

Among the 69 *Botryosphaeriaceae* isolates included in our study, the RNA virome of 15 of them was characterized through the HTS of purified dsRNAs, comprising 4 isolates of *D. seriata*, 4 isolates of *D. mutila*, 3 isolates of *N. parvum*, 2 isolates of *S. viticola*, and 1 isolate each of *L. viticola* and *D. intermedia* (Table 1). For the 15 *Botryosphaeriaceae* isolates sequenced by HTS, growth data at 28 °C and necrosis length were determined, and the results are provided in Table 1. As expected, the growth of the different isolates varied between isolate and species. The strategy of sequencing purified dsRNA allowed us to identify RNA viruses, including dsRNA and single-stranded (ss) RNA viruses, as dsRNA molecules are replicative forms of viruses with ss RNA genomes [60,61]. This approach does not allow for the identification of DNA viruses.

After quality trimming and demultiplexing, the reads were submitted to a de novo assembly, and the resulting contigs were annotated via BlastN and BlastX analysis against the GenBank database using a conservative 10^−3^ e-value cut-off. For nine isolates (9/15, 60%), no viral contigs could be identified, suggesting that the corresponding fungal isolates were very likely virus-free (Table 1). In contrast, viral contigs could be identified from the remaining six isolates (Table 2). Some contigs showed significant identity with five *Botryosphaeriaceae* viruses already described in previous studies [44,45,46]. Diplodia seriata endornavirus 1 (DsEV1), initially described from a *D. seriata* isolate from an Esca symptomatic vine [44], was detected in two isolates of *D. mutila* (LAG01 and BRA08). Indeed, the two reconstructed scaffolds (10,127 nt and 9848 nt, respectively) showed 89.2% nt identity with the reference isolate of DsEV1 (GenBank accession number MK584822), above the species demarcation threshold accepted for the *Betaendornavirus* genus (75% nt identity) [62]. The two RNA segments of Diplodia seriata partitivirus 1 (DsPV1), previously characterized from a single loquat (*Eriobotrya japonica*) isolate of *D. seriata* [47], were reconstructed from the HTS data obtained for *D. mutila* BRA08 and *L. viticola* LAG05. The RNA1-deduced RNA-dependent RNA polymerase (RdRp) sequences showed, respectively, 94.2% and 93.6% aa identity with that of the reference isolate (UOK20169), above the threshold for species demarcation in the *Partitiviridae* family (90% aa identity in the RdRp) [63]. Finally, three contigs having homology with known mycoviruses were reconstructed in the *N. parvum* PER20 isolate: One showed 95% nt identity with Neofusicoccum parvum narnavirus 3 (NpNV3), previously identified in a grapevine *N. parvum* isolate (MW175883, [45]); the second one shared 89.1% nt identity with Neofusicoccum parvum victorivirus 1 (NpVV1), characterized from the same *N. parvum* isolate (MW175879, [45]); and the last one displayed 97.8% nt identity with the negative-sense RNA virus Alternaria tenuissima negative-strand RNA virus 1 (AtNsRV1), which was first detected in an *Alternaria tenuissima* isolate from grapevine (NC_076392, [44]). 

In contrast, some contigs reconstructed from the *D. seriata* BoF981, *D. mutila* ARB45, and *N. parvum* PER20 isolates (Table 2) showed only distant relationships with members of the *Bunyavirales* (at best, 45% aa identity with RdRp of Macrophomina phaseolina mycobunyavirus 1 (GenBank accession number QOE55579)) or belonged to the family *Narnaviridae* (55% aa identity with the RdRp of Monilinia narnavirus H (GenBank accession number QED42934)) and the proposed Fusagraviridae family (70% aa identity with the RdRp of Diplodia scrobiculata RNA virus 1 (GenBank accession number YP003359178)). These viral contigs were then assembled into scaffolds and extended through successive rounds of mapping of residual reads using CLC GW to yield finalized contigs spanning at least the entire potential coding of the corresponding viral genomes.

### 3.2. Molecular Features and Phylogenetic Relationships of the Identified Novel Viruses

#### 3.2.1. A New Fusagravirus in *D. mutila* and *N. parvum*

**Figure 1:**
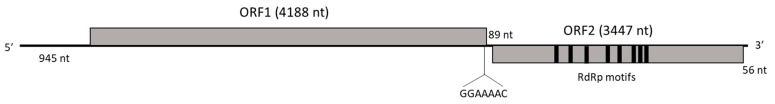
Schematic representation of the genomic organization of Diplodia mutila fusagravirus 1 (isolate ARB45). The length of the 5′ noncoding region (NCR), 3′ NCR, intergenic region, and open reading frames (ORFs) are indicated. The eight conserved motifs in the ORF2-coded RNA-dependent RNA polymerase (RdRp) are shown as thick black stripes. The position of the candidate shifty heptamer (GGAAAAC) is also indicated.

Two scaffolds with a distant identity to members of the proposed Fusagraviridae family were reconstructed from the reads from *D. mutila* ARB45 and *N. parvum* PER20. The genome sequence of the ARB45 isolate was completed through 5′ and 3′ RACE experiments using the primers designed from the scaffold sequence (Table S1). The ARB45 complete genome is 8725 nt long and encodes two large open reading frames (ORFs) of, respectively, 4188 nt and 3447 nt. The 5′ noncoding region (NCR) is 945 nt long, while the 3′ NCR has a length of 56 nt. No further efforts were made to complete the PER20 isolate genome, which comprises 8185 nt and potentially encodes the ORFs of the same length and shows an overall nucleotide identity of 77.9% with the ARB45 isolate. The genomic organization is comparable with that of other fusagraviruses (Figure 1). The ORF1 potentially encodes a protein of 1396 aa, showing at best only 21.3% aa identity with the hypothetical protein 1 of Fusarium poae dsRNA virus 3 in the proposed family Fusagraviridae [64]. On the other hand, the ORF2-encoded protein (1149 aa) showed at best 69.9% aa identity with the RdRp of Diplodia scrobiculata RNA virus 1 (DsRV1) [65] (Table S2). Nevertheless, the relationships between DsRV1 and the new virus are tenuous, with some marked differences, such as the length of the genome, the 5′ NCR, and ORF1. Interestingly, as for most fusagraviruses except for DsRV1 [62], a candidate shifty heptamer (GGAAAAC) was found in the sequence of the ARB45 and PER20 isolates, located immediately before the ORF1 UAA stop codon, which could mediate programmed –1 ribosomal frameshifting (–1PRF) [66]. Moreover, the eight conserved motifs in the RdRp of dsRNA viruses [67] were found in the ORF2-deduced protein (Figure 1). These characteristics strongly suggest that this fusagravirus-like agent is a novel species in the proposed Fusagraviridae family, for which the name Diplodia mutila fusagravirus 1 (DmFV1) is proposed, with two isolates detected in *D. mutila* ARB45 and *N. parvum* PER20, sharing 90.1% and 92.1% aa identities in their ORF1- and ORF2-deduced proteins, respectively (Table S2). The two genomic sequences have been deposited in GenBank under accession numbers ON236579 and ON236578, respectively (Table 2). 

**Figure 2:**
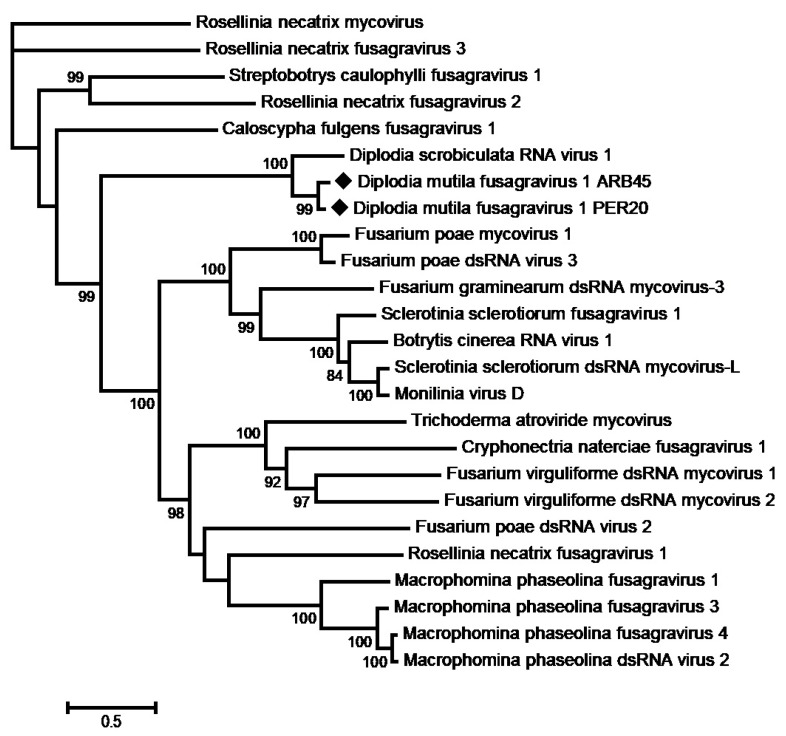
Phylogenetic analysis based on the alignment of RNA-dependent RNA polymerase sequences from fusagraviruses. The maximum likelihood tree with the highest log likelihood is shown. The percentage of trees in which the associated taxa clustered together is shown next to the branches. The tree is drawn to scale, with branch lengths measured in the number of substitutions per site. Sequences generated from this study are indicated with black diamonds. Bootstrap values below 70% were removed.

The phylogenetic tree reconstructed from the alignment of the RdRp sequences from members of the proposed Fusagraviridae family (Figure 2) showed that DmFV1 clustered in a separate clade comprising Rosellinia necatrix mycovirus, Rosellinia necatrix fusagravirus 2, Rosellinia necatrix fusagravirus 3, Streptobotrys caulophylli fusagravirus 1, Caloscypha fulgens fusagravirus 1, and DsRV1. A phylogenetic tree based on the ORF1-deduced protein showed a comparable clustering, with the exception of DsRV1, as explained above (Figure S1). 

#### 3.2.2. A New Mycobunyavirus in *D. seriata*

**Figure 3:**
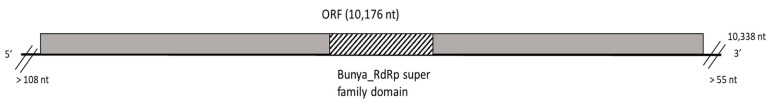
Schematic representation of the genomic organization of Diplodia seriata mycobunyavirus 1. The minimal length of the 5′ and 3′ NCRs are indicated as well as the length of the open reading frame (ORF). The conserved Bunyavirus RNA-dependent RNA polymerase (RdRp) motif (cl20265, E-value 1.16 × 10^6^) is shown in the ORF1-deduced protein with diagonal stripes.

**Figure 4:**
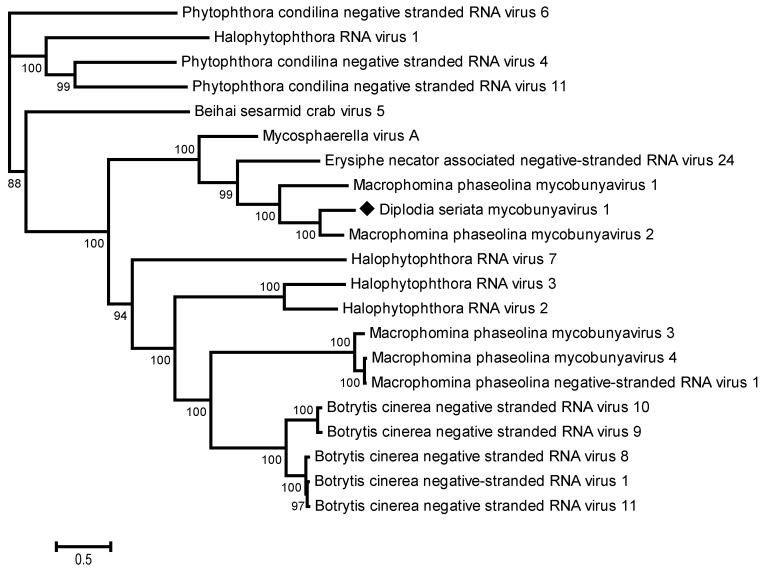
Phylogenetic analysis based on the alignment of RNA-dependent RNA polymerase sequences from putative Mycobunyaviridae members and unassigned negative-sense-stranded RNA viruses. The maximum likelihood tree with the highest log likelihood is shown. The percentage of trees in which the associated taxa clustered together is shown next to the branches. The tree is drawn to scale, with branch lengths measured in the number of substitutions per site. The sequence generated from this study is indicated with a black diamond. Bootstrap values below 70% were removed.

From 1,314,206 total reads from the *D. seriata* isolate BoF981, a contig of 10,339 nt was reconstructed, integrating 7.2% of the total reads (Table 2). The BlastN analysis showed homology with the RdRp genes of Macrophomina phaseolina mycobunyavirus 2 (72%, MpMBV2 partial sequence, GenBank accession number MT062422) and Macrophomina phaseolina mycobunyavirus 1 (67% nt identity, MpMBV1, GenBank accession number MT062421) [68]. The contig harbors a large ORF of 10,176 nt, encoding a putative RdRp that contains a Bunya_RdRp domain (clc20265) (Figure 3). Pairwise comparisons between RdRp sequences of various members of the proposed Mycobunyaviridae family [69] revealed the RdRp of the new virus to share 77% identity with MpMBV2 RdRp (partial sequence restricted to 686 aa) and only 45% aa identity with the complete MpMBV1 RdRp, and even more distant identity levels with other negative-sense-stranded RNA viruses belonging to the *Bunyavirales* order. These results suggest that the *D. seriata* mycobunyavirus represents a novel species for which the name Diplodia seriata mycobunyavirus 1 (DsMBV1) is proposed here. The phylogenetic analysis performed using the RdRp sequences of various mycobunyaviruses and unassigned negative-strand RNA viruses showed that the DsMBV1 clustered with mycoviruses likely belongs to the proposed family Mycobunyaviridae, such as MpMBV1 and MpMBV2 [68,69], which confirms their taxonomical relationships (Figure 4). 

#### 3.2.3. A New Narnavirus in *D. seriata*

**Figure 5:**
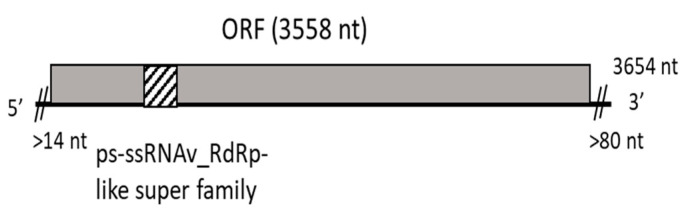
Schematic representation of the genomic organization of Diplodia seriata narnavirus 1. The minimal length of the 5′ and 3′ NCRs are indicated, as well as the length of the open reading frame (ORF). The conserved catalytic core domain of RNA-dependent RNA polymerase (RdRp) from the positive-sense single-stranded RNA viruses (cl40470, E-value 9.16 × 10^4^) is shown in the ORF-deduced protein with diagonal stripes.

**Figure 6:**
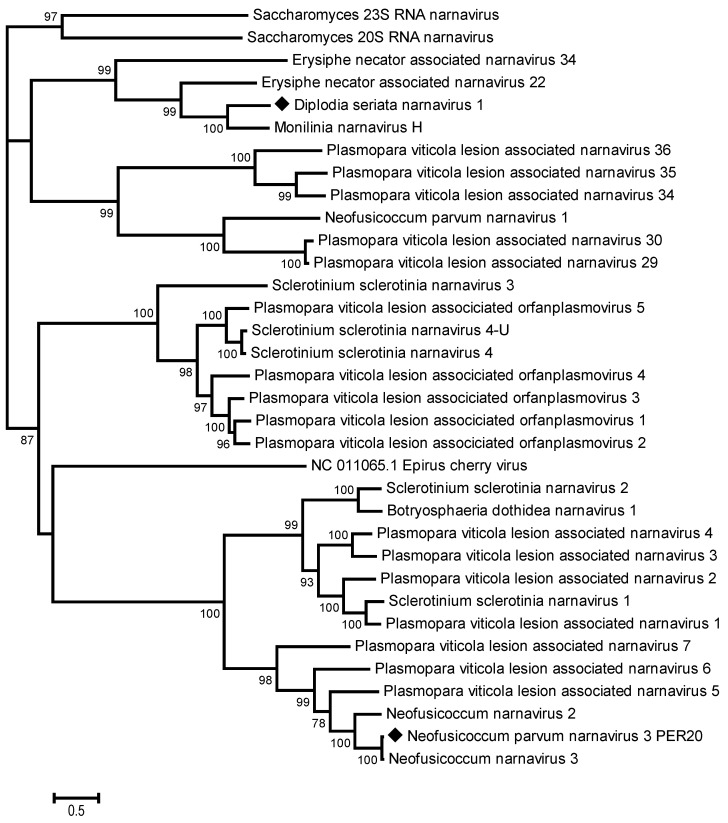
Phylogenetic analysis based on the alignment of RNA-dependent RNA polymerase sequences from selected narnaviruses. The maximum likelihood tree with the highest log likelihood is shown. The percentage of trees in which the associated taxa clustered together is shown next to the branches. The tree is drawn to scale, with branch lengths measured in the number of substitutions per site. Sequences generated from this study are indicated with black diamonds. Members of the *Narnaviridae* family are indicated, as well as the proposed Polynarnaviridae family. Bootstrap values below 70% were removed. Epirus cherry virus (*Botourmiaviridae* family) was used as the outgroup.

From the same BoF81 isolate of *D. seriata* infected by DsMBV1, a second viral contig of 3654 nt was identified, showing distant nt and aa identities with some members of the *Narnaviridae* family. No further efforts were made to complete this genomic sequence, but the contig encodes a unique ORF of 3558 nt, encoding a putative RdRp of 1186 aa, with the conserved catalytic core domain of an RdRp of positive-sense, single-stranded RNA viruses located between aa 600 and 720 (Figure 5). The phylogenetic tree based on the RdRp alignment of narna-like viruses (Figure 6) showed that this virus clustered together with *Narnaviridae* members, the most closely related virus being Monillinia narnavirus H (54.3% aa identity). This suggests that the complete coding potential of the corresponding virus was determined and that it represents a novel species in the *Narnaviridae* family, named Diplodia seriata narnavirus 1 (DsNV1). 

### 3.3. Distribution of Mycoviruses within a Collection of Botryosphaeriaceae Isolates, and Analysis of the Genetic Diversity of the Identified Viruses

**Figure 7:**
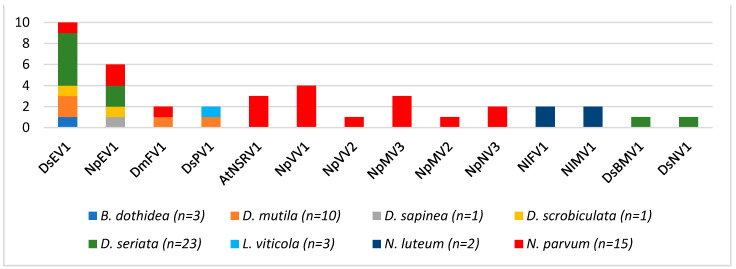
Virus distribution in the different strains of *Botryosphaeriaceae* species analyzed.

The 69 *Botryosphaeriaceae* isolates (Table 1) were screened for the presence of not only the eight mycoviruses identified in the 15 isolates analyzed by HTS in this study but also for other grapevine-associated *Botryosphaeriaceae*-infecting mycoviruses described in the literature [44,45,54,58]. Altogether, the presence and the genetic diversity of 20 *Botryosphaeriaceae*-infecting mycoviruses were thus investigated. The 20 investigated mycoviruses are double-stranded RNA viruses (*n* = 5), or positive-sense single-stranded (*n* = 12) and negative-sense single-stranded (*n* = 2) viruses, representing 11 various families, namely Fusagraviridae (DmFV1), *Totiviridae* (NpVV1 and NpVV2), *Partitiviridae* (DsPV1), *Chrysoviridae* (NpCV1), *Narnaviridae* (NpNV1, NpNV2, NpNV3, and DsNV1), *Mitoviridae* (NpMV1, NpMV2, NpMV3, and NlMV1), *Endornaviridae* (DsEV1 and NpEV1), *Fusariviridae* (NlFV1), *Botourmiaviridae* (NpOulV1), *Mymonaviridae* (AtNsRV1), *Bunyavirales* (DsMBV1), and an unclassified +ssRNA (NpVlV1) (Table S1). As shown in Table 1, none of the 20 tested viruses was identified from the isolates belonging to five species (*S. viticola*, *N. ribis*, *L. pseudothreobromae*, *D. intermedia*, and *D. rosulata*). Mycoviruses were detected in the remaining species but still with a high proportion of isolates free of the tested viruses, comprising between 60% (*N. parvum*) and 70% (*D. mutila*). Nevertheless, some isolates of *N. parvum*, *N. luteum*, *D. mutila*, and *D. seriata* showed a complex RNA mycovirome, comprising several coinfecting viruses (Table 1, Figure 7). Among the 20 mycoviruses included in this survey, 6 were not detected within the collection, namely NpNV1, NpNV2, NpMV1, NpCV1, NpOulV1, and NpVlV1, previously characterized from the *N. parvum* isolates from asymptomatic grapevine plants [44]. In contrast, the remaining 14 mycoviruses were detected in at least one isolate. As shown in Figure 7, most of these mycoviruses (10/14) were detected specifically in a single species, namely NlFV1 and NlMV1 in *N. luteum*; DsBMV1 and DsNV1 in *D. seriata*; and NpVV1, NpVV2, NpNV3, NpMV2, NpMV3, and AtNRSV1 in *N. parvum*, even though AtNRSV1 had previously been characterized from *Alternaria tenuissima* [44]. By contrast, the remaining four mycoviruses were detected in several species and even several genera. DsPV1, initially detected in *D. seriata* [47], was found in this study in some isolates of *D. mutila* and *L. viticola.* The novel DmFV1 was characterized using the HTS data in *D. mutila* and *N. parvum*. Finally, the two endornaviruses (DsEV1 and NpEV1) were, respectively, detected in five and four *Botryosphaeriaceae* species that belonged to different genera such as *Botryosphaeria*, *Diplodia*, and *Neofusicoccum.* It is noteworthy that these viruses were most prevalent in *D. seriata.* In total, this screening allowed us to conclude that one-third (23/69) of the *Botryosphaeriaceae* isolates were infected by at least one of the fourteen mycoviruses detected in the collection. A significant proportion of isolates (10/23, 43.5%) were found to be coinfected by several viruses, with very complex RNA mycoviromes for some *N. parvum* isolates (COLB and PER20, Table 1). 

In order to evaluate the genetic variability of the detected mycoviruses, PCR amplicons were sequenced. Intraspecies genetic variability as well as phylogenetic affinities between isolates are shown in Table 3 and Figure S2a–g. Globally, the level of nt variability observed in the short amplified fragments was notable, with a maximum nt divergence of 12.5% observed between the two isolates of DsEV1 but only 1.9% aa divergence for the encoded protein, thus clearly placing all isolates within the same species. No clustering based on the *Botryosphaeriaceae* host species could be observed (Figure S2b,c,e).

Nevertheless, the genetic variability observed in many cases between amplicons of the same virus from different fungal isolates rules out the possibility of PCR contamination. 

## 4. Discussion

*Botryosphaeriaceae*, which are generally hemibiotrophic, are distributed worldwide, infect a large number of hosts, and are often associated with diseases on woody species of agronomic or forestry interest [70]. So far, only a few fungal species belonging to the family *Botryosphaeriaceae* have been examined for the presence of mycoviruses: *B. dothidea*, *N. parvum, N. luteum, D. scrobiculata*, and *D. seriata*. Here, we report mycovirus screening in several additional *Botryosphaeriaceae* species, including *L. viticola*, *S. viticola*, *D. mutila*, and *D. intermedia*, as well as the analysis of additional isolates of *N. parvum* and *D. seriata*. In total, fifteen isolates from six *Botryosphaeriaceae* species were submitted to mycovirus screening using a dsRNA-based HTS approach. Two isolates of *S. viticola* and a single isolate of *D. intermedia* were found to be virus-free, as well as some *D. seriata* isolates (3/4), *N. parvum* (2/3), and *D. mutila* (1/4). Due to the dsRNA-based HTS strategy we followed, we cannot exclude the possibility that some DNA viruses, known to be more difficult to detect using this technique, could infect those isolates, even if there seem to be only very few DNA viruses infecting fungi so far [22]. The remaining six mycelia analyzed were found to be infected by a total of eight mycoviruses. Three of them—DmFV1, DsMBV1, and DsNV1—correspond to novel species in the proposed families Fusagraviridae, Mycobunyaviridae, and the *Narnaviridae* family, respectively. The other five mycoviruses have already been described in previous studies (DsPV1, DsEV1, NpVV1, NpNV3, and AtNsRV1), sometimes from a different fungal host, such as DsEV1 and DsPV1, which had been originally described from *D. seriata* [44,47] and were detected here in two *D. mutila* isolates (DsEV1), and in one isolate of *D. mutila* and of *L. viticola* (DsPV1). These findings of the same mycoviral species in different host species raise questions about mycoviruses’ host range and transmission mechanisms. 

A large collection of *Botryosphaeriaceae* isolates was then screened for infection by mycoviruses in order to provide some clues to these questions. Besides the 8 viruses identified by HTS, 12 other mycoviruses previously identified in *Botryosphaeriaceae* isolated from grapevine [44,45,54,58] were included in this study, resulting in the PCR screening of 20 mycoviruses, belonging to 11 viral families. Six mycoviruses originally identified by Nerva et al. [44] in *Botryosphaeriaceae* isolates from Italian grapevines were not detected in our collection, probably reflecting some geographical specificity of the mycovirome, as was previously observed for the virome of *Rosellinia necatrix* isolates from Israel and Spain [71]. 

Based on PCR screening results, five species (*S. viticola*, *N. ribis*, *L. pseudothreobromae*, *D. intermedia*, and *D. rosulata*) were found to be infected by none of the viruses tested. However, these species were only represented by a limited number of isolates (often one to three). Also, we cannot exclude the possibility of the nongenericity of the primers designed and/or used for the screening and that some viral isolates may have thus escaped detection. Conversely, although unlikely in light of the literature, it cannot be ruled out that positive detections may result from the integration of viral genomic segments in the host genome. Our screening data suggest that some mycoviruses are probably specialists, with a host range restricted to a single species, while others are more generalists and were detected in several species and even in members of several genera. These data are consistent with an increasing number of studies indicating that some mycoviruses have a relatively wide host range [71,72,73,74,75]. These results contradict previous notions, as it was previously believed that specificity for a given host species was the rule for mycoviruses [76]. The most prevalent mycoviruses within our collection are the two *Endornaviridae* members, DsEV1 (genus *Betaendornavirus*) and NpEV1 (genus *Alphaendornavirus*), which were detected in 14.5% (10/69) and 8.7% (6/69) of isolates, within five species (three genera), and four species (two genera), respectively. In order to assess the intraspecific variability of each of the viruses detected in more than one fungal isolate, the short PCR products generated during the screening were sequenced. No clustering of viral isolates according to the fungal host was identified, suggesting that there was no co-speciation between viruses and their fungal hosts, a conclusion that must be tempered by the limited number of mycoviruses involved in the present analysis. These results are nevertheless in line with the examination of co-phylogeny of viruses and their hosts performed by Myers and James [77], which suggest recurrent, even occasional, host shifts. Taken together, these results suggest the possibility of exchanges of mycoviruses between fungal hosts. Several studies already proposed that cross-species transmission may occur in nature, even between phylogenetically distant fungal species, probably during the coinfection of the same plant [74,75]. Even if infrequent and inefficient, these cross-species transmission events contradict the dogma that mycoviruses are only transmitted horizontally between vegetatively compatible fungi through hyphal anastomosis. A few studies have been conducted to explore the factors involved in such vegetative compatibility-independent transmission (for review, see [23,77]). Some viruses appear to be able to weaken the vegetative incompatibility system, such as Sclerotinia sclerotiorum mycoreovirus 4, which downregulates the genes involved in nonself-recognition pathways, thus facilitating horizontal transmission of heterologous mycoviruses [78]. Some authors also suggest that some mycoviruses may persist in the environment and can be infectious when applied extracellularly to their hosts, as shown for the DNA mycovirus Sclerotinia sclerotiorum hypovirulence-associated DNA virus 1 [79,80]. This mycovirus was also shown to infect a mycophagous insect (*Lycoriella ingenua*) that it then uses as a vector for its transmission [81], raising the question of the significance of insect-mediated pathways in mycoviral transmission. 

The HTS-based RNA mycovirome screening showed that the majority (60%, 9/15) of the *Botryosphaeriaceae* isolates included in this work are RNA-virus-free. The PCR-based screening allowed us to extend this analysis to a broader collection of *Botryosphaeriaceae* isolates obtained in a large majority from grapevine. Only 33.3% (23/69) of the isolates were found to be infected by at least one of the twenty mycoviruses tested, a value close to that obtained by HTS. However, the rate of infection varied significantly between fungal species, in line with previous studies on various pathosystems [82]. A significant proportion of isolates (10/69, 14.5%) were found to be coinfected by several viruses, sometimes resulting in a complex mycovirome. Because of this significant coinfection rate and the relatively small proportion of the infected isolates, it proved very challenging to attribute phenotypic differences to the presence of a single virus. Moreover, it is known that in cases of coinfection, the interplay between viruses may result in various and even opposite interactions [24]. Bearing these limitations in mind, the possible effects of each virus on host life history traits could be examined with additional experiments to better characterize the potential impacts on host biology of some of the mycoviruses identified here. One of the possibilities would be to have access to a larger number of isolates of the same species obtained from the same plots, which would in theory allow us to average out the contribution of the genetic makeup of fungal isolates. However, due to the differences in the genetic backgrounds of host isolates, and the fact that such variations in mycovirus isolates can also influence the final phenotype [83], the best strategy to evaluate the impact of mycoviral infection on host phenotype would be to conduct comparative studies using infected fungal isolates and the corresponding isogenic virus-free isolates. 

With the aim of developing biocontrol strategies using mycoviruses, many studies have so far focused on analyzing fungal isolates with strong hypovirulent phenotypes (for review, see [20,22]), without considering the whole mycovirome. Some elements highlighted in our study, such as the coinfection rate and the potential for cross-species transmission, should lead us to take caution since, as already pointed out by some authors [74,83], the outcome of the interactions between host and mycoviruses, and even between mycoviruses themselves during coinfections, still carry significant unpredictability. 

## Figures and Tables

**Table 1:** List of *Botryosphaeriaceae* isolates included in the present study and viruses detected by either PCR or HTS analysis. Isolates in bold were analyzed by HTS of double-stranded RNA. The length of necrosis and growth at 28 °C (AUC) for the 15 isolates analyzed by dsRNA-HTS are provided.

Genus	Species	Isolate	Country	Region	Host	Cultivar	Sampling Date	Symptoms	Sample Localization	AUC at 28 °C **	Necrosis Length (mm)	Viruses
*Botryosphaeria*	*B. dothidea*	CBS110302 *	Portugal	Montemor-o-Novo	*V. vinifera*	na	1996	na	na	na	na	0
*Botryosphaeria*	*B. dothidea*	LAT32	France	New Aquitaine	*V. vinifera*	Cabernet franc	2008	S	N	na	na	DsEV1
*Botryosphaeria*	*B. dothidea*	OGE14	France	Champagne	*V. vinifera*	Pinot noir	2008	S	B	na	na	0
*Diplodia*	*D. intermedia*	BEI06	France	Burgundy	*V. vinifera*	Chardonnay	2008	S	B	na	na	0
***Diplodia***	***D. intermedia***	**BEI39**	**France**	**Burgundy**	***V. vinifera***	**Chardonnay**	**2008**	**H**	**B**	**819**	**7.3**	**0**
*Diplodia*	*D. mutila*	ARB07	France	Jura	*V. vinifera*	Trousseau	2008	S	B	na	na	0
*Diplodia*	*D. mutila*	ARB44	France	Jura	*V. vinifera*	Trousseau	2008	H	B	na	na	0
***Diplodia***	***D. mutila***	**ARB45**	**France**	**Jura**	***V. vinifera***	**Trousseau**	**2008**	**H**	**B**	**245**	**2.75**	**DmFV1**
***Diplodia***	***D. mutila***	**BEI36**	**France**	**Burgundy**	***V. vinifera***	**Chardonnay**	**2008**	**H**	**B**	**330**	**3.67**	**0**
***Diplodia***	***D. mutila***	**BRA08**	**France**	**Champagne**	***V. vinifera***	**Pinot noir**	**2008**	**S**	**B**	**1150**	**8.6**	**DsPV1 DsEV1**
*Diplodia*	*D. mutila*	CBS112553 *	Portugal	Montemor-o-Novo	*V. vinifera*	na	1997	na	na	na	na	0
*Diplodia*	*D. mutila*	GRA09	France	New Aquitaine	*V. vinifera*	Ugni blanc	2008	S	N	na	na	0
***Diplodia***	***D. mutila***	**LAG01**	**France**	**New Aquitaine**	***V. vinifera***	**Cabernet Sauvignon**	**2008**	**S**	**N**	**928**	**13.3**	**DsEV1**
*Diplodia*	*D. mutila*	LAG27	France	New Aquitaine	*V. vinifera*	Cabernet Sauvignon	2008	S	N	na	na	0
*Diplodia*	*D. mutila* ^1^	CBS43.182 *	Netherlands	Maarseveen	*Fraxinus excelsior*	na	1982	na	na	na	na	0
*Diplodia*	*D. rosulata*	CBS116470 *	Ethiopia	na	*Prunus africana*	na	2001	na	na	na	na	0
*Diplodia*	*D. sapinea*	CBS109725 *	South Africa	Habinsaran	*Pinus patula*	na	2001	na	na	na	na	NpEV1
*Diplodia*	*D. scrobiculata*	CBS118110 *	USA	Wisconsin	*Pinus banksiana*	na	na	na	na	na	na	NpEV1 DsEV1
*Diplodia*	*D. seriata*	ARB01	France	Jura	*V. vinifera*	Trousseau	2008	S	B	na	na	0
*Diplodia*	*D. seriata*	ARB18	France	Jura	*V. vinifera*	Trousseau	2008	S	N	na	na	0
*Diplodia*	*D. seriata*	BEI03	France	Burgundy	*V. vinifera*	Chardonnay	2008	S	N	na	na	0
*Diplodia*	*D. seriata*	BEI25	France	Burgundy	*V. vinifera*	Chardonnay	2008	S	N	na	na	0
*Diplodia*	*D. seriata*	BoF00-14	France	Champagne	*V. vinifera*	Pinot Meunier	2000	S	na	na	na	NpEV1
*Diplodia*	*D. seriata*	BoF00-5	France	New Aquitaine	*V. vinifera*	Cabernet Sauvignon	2000	S	N	na	na	0
***Diplodia***	***D. seriata***	**BoF98-1**	**France**	**Languedoc Roussillon**	***V. vinifera***	**Syrah**	**1998**	**S**	**na**	**945**	**9.82**	**DsBMV1 DsNV1**
*Diplodia*	*D. seriata*	BoF99-7	France	Rhône Valley	*V. vinifera*	Clairette	1999	S	na	na	na	NpEV1
*Diplodia*	*D. seriata*	BoF99-8	France	Languedoc Roussillon	*V. vinifera*	Syrah	1999	S	na	na	na	DsEV1
***Diplodia***	***D. seriata***	**BRA16**	**France**	**Champagne**	***V. vinifera***	**Pinot noir**	**2008**	**S**	**B**	**1306**	**6.8**	**0**
*Diplodia*	*D. seriata*	CBS112555 *	Portugal	Montemor-o-Novo	*V. vinifera*	na	1997	na	na	na	na	0
*Diplodia*	*D. seriata*	IRA 21	France	Burgundy	*V. vinifera*	Pinot noir	2008	S	B	na	na	DsEV1
*Diplodia*	*D. seriata*	IRA33	France	Burgundy	*V. vinifera*	Pinot noir	2008	S	B	na	na	DsEV1
*Diplodia*	*D. seriata*	LAG13	France	New Aquitaine	*V. vinifera*	Cabernet Sauvignon	2008	S	N	na	na	0
***Diplodia***	***D. seriata***	**LAT16**	**France**	**New Aquitaine**	***V. vinifera***	**Cabernet franc**	**2008**	**S**	**N**	**1182**	**5.03**	**0**
***Diplodia***	***D. seriata***	**LAT28**	**France**	**New Aquitaine**	***V. vinifera***	**Cabernet franc**	**2008**	**S**	**B**	**1292**	**5.63**	**0**
*Diplodia*	*D. seriata*	MOT02	France	Burgundy	*V. vinifera*	Chardonnay	2008	S	N	na	na	0
*Diplodia*	*D. seriata*	PER01	France	Champagne	*V. vinifera*	Chardonnay	2008	S	B	na	na	DsEV1
*Diplodia*	*D. seriata*	PLU03	France	Champagne	*V. vinifera*	Chardonnay	2008	S	N	na	na	0
*Diplodia*	*D. seriata*	ROM14	France	Champagne	*V. vinifera*	Chardonnay	2008	S	B	na	na	DsEV1
*Diplodia*	*D. seriata*	ROU03	France	Alsace	*V. vinifera*	Gewurztraminer	2008	S	B	na	na	0
*Diplodia*	*D. seriata*	TUR16	France	Alsace	*V. vinifera*	Auxerrois	2008	S	N	na	na	0
*Diplodia*	*D. seriata*	VIE51	France	Champagne	*V. vinifera*	Chardonnay	2008	H	B	na	na	0
*Lasiodiplodia*	*L. pseudotheobromae*	CBS116459 *	Costa Rica	San Carlos	*Gmelina arborea*	na	na	na	na	na	na	0
*Lasiodiplodia*	*L. pseudotheobromae*	CBS116460 *	Costa Rica	San Carlos	*Acacia mangium*	na	na	na	na	na	na	0
*Lasiodiplodia*	*L. viticola*	CBS128313 *	USA	Arkansas	*V. vinifera*	Vignoles	na	S	na	na	na	0
***Lasiodiplodia***	***L. viticola***	**LAG05**	**France**	**New Aquitaine**	***V. vinifera***	**Cabernet Sauvignon**	**2008**	**S**	**H-p**	**1336**	**23.30**	**DsPV1**
*Lasiodiplodia*	*L. viticola*	LAG78	France	New Aquitaine	*V. vinifera*	Cabernet-Sauvignon	2008	S	H-p	na	na	0
*Neofusicoccum*	*N. luteum*	CAP37	Portugal	na	*V. vinifera*	na	na	na	na	na	na	NlMV1 NlFV1
*Neofusicoccum*	*N. luteum*	CBS110299 *	Portugal	Oeiras	*V. vinifera*	na	1996	na	na	na	na	NlMV1 NlFV1
*Neofusicoccum*	*N. parvum*	ALI03	France	Languedoc Roussillon	*V. vinifera*	Sauvignon	2008	S	N	na	na	AtNSRV1 NpVV1
*Neofusicoccum*	*N. parvum*	ALI30	France	Languedoc Roussillon	*V. vinifera*	Sauvignon	2008	H	N	na	na	NpVV1 NpMV3 DsEV1
***Neofusicoccum***	***N. parvum***	**AUD25**	**France**	**Languedoc Roussillon**	***V. vinifera***	**na**	**2008**	**S**	**H-p**	**1093**	**13.62**	**0**
*Neofusicoccum*	*N. parvum*	AUD31	France	Languedoc Roussillon	*V. vinifera*	na	2008	S	N	na	na	0
*Neofusicoccum*	*N. parvum*	BdF00-14	France	New Aquitaine	*V. vinifera*	Merlot	2000	S	na	na	na	0
*Neofusicoccum*	*N. parvum*	BdF00-21	France	Champagne	*V. vinifera*	na	2000	S	N	na	na	0
*Neofusicoccum*	*N. parvum*	BdF00-3	France	Languedoc Roussillon	*V. vinifera*	Syrah	2000	S	na	na	na	0
*Neofusicoccum*	*N. parvum*	BdF00-8	France	New Aquitaine	*V. vinifera*	Merlot	2000	S	N	na	na	0
*Neofusicoccum*	*N. parvum*	CBS110301 *	Portugal	na	*V. vinifera*	na	1996	-	na	na	na	0
*Neofusicoccum*	*N. parvum*	COLB	France	Burgundy	*V. vinifera*	Chardonnay	2009	S	na	na	na	NpNV3 NpEV1 NpMV2 NpMV3 NpVV1 NpVV2
*Neofusicoccum*	*N. parvum*	COU02	France	New Aquitaine	*V. vinifera*	Cabernet Sauvignon	2008	S	N	na	na	NpMV3 NpEV1
*Neofusicoccum*	*N. parvum*	PER04	France	Champagne	*V. vinifera*	Chardonnay	2008	S	B	na	na	AtNSRV1
***Neofusicoccum***	***N. parvum***	**PER20**	**France**	**Champagne**	***V. vinifera***	**Chardonnay**	**2008**	**S**	**N**	**1717**	**19.22**	**AtNSRV1 NpVV1 DmFV1 NpNV3**
*Neofusicoccum*	*N. parvum*	SAI07	France	Burgundy	*V. vinifera*	Ugni blanc	2008	S	N	na	na	0
***Neofusicoccum***	***N. parvum***	**VIE35**	**France**	**Champagne**	***V. vinifera***	**Chardonnay**	**2008**	**S**	**H-p**	**1665**	**33.70**	**0**
*Neofusicoccum*	*N. ribis*	CBS114472 *	Hawaii	na	*Leucadron Safari Sunset*	na	1998	na	na	na	na	0
*Neofusicoccum*	*N. ribis*	CBS115475 *	USA	New York	*Ribes sp.*	na	1998	na	na	na	na	0
*Spencermartinsia*	*S. viticola*	CBS117009 *	Spain	Catalonia	*V. vinifera*	Garnatxa negra	2004	na	na	na	na	0
***Spencermartinsia***	***S. viticola***	CBS121000 *	**USA**	**California**	***V. vinifera***	**Cabernet Sauvignon**	**2008**	**na**	**na**	**666**	**8**	**0**
***Spencermartinsia***	***S. viticola***	**GAR09**	**France**	**Languedoc-Roussillon**	***V. vinifera***	**Sauvignon**	**2008**	**S**	**B**	**276**	**2.39**	**0**
*Spencermartinsia*	*S. viticola*	GAR47	France	Languedoc Roussillon	*V. vinifera*	Sauvignon	2008	H	B	na	na	0

* This indicates isolates from the Westerdijk Institute; ^1^ newly renamed *Diplodia fraxini*; ** AUC: area under the curve; B: bark; N: necrosis; na: not available; H: healthy plant; S: symptomatic plant; H-p: healthy part.

**Table 2:** Overview of the identified viruses with corresponding data on Illumina dsRNA sequencing for each fungal isolate. Tentative novel viruses are in bold.

Species	Isolate	Virus	Total Reads	Mapped Reads (% of Total Reads)	Average Coverage	Contig Length (nt)	Predicted Protein Encoded ^1^	Accession Number
*D. mutila*	ARB45	**DmFV1**	446,665	347,375 (77.8%)	4905	8725	HP, RdRp	ON236579 ^2^
*D. mutila*	BRA08	DsPV1 RNA1	1,499,218	82,833 (5.5%)	12,068	1529	RdRp	ON236584 ^3^
		DsPV1 RNA2		50,293 (3.4%)	6759	1456	CP	ON236585 ^3^
		DsEV1		875,766 (58.4%)	52,996	9848	Polyprotein	ON236581 ^3^
*D. mutila*	LAG01	DsEV1	1,313,885	1,103,013 (84%)	13,199	10,127	Polyprotein	ON236580 ^2^
*L. viticola*	LAG05	DsPV1 RNA1	958,446	324,630 (33.9%)	52,996	1338	RdRp	ON236582 ^3^
		DsPV1 RNA2		109,971 (11.5%)	15,666	1449	CP	ON236583 ^3^
*N. parvum*	PER20	NpVV1	1,302,602	845 (0.1%)	29	5188	CP, RdRp	ON236575 ^3^
		**DmFV1**		112,024 (8.6%)	2909	8185	HP, RdRp	ON236578 ^3^
		NpNV3		1258 (0.1%)	130	2071	RdRp	ON236576 ^3^
		AtNsRV1		254,239 (19.5%)	6356	8921	RdRp	ON236577 ^3^
*D. seriata*	BoF981	**DsMBV1**	1,314,206	94,816 (7.2%)	2064	10,339	RdRp	ON236586 ^3^
		**DsNV1**		9504 (0.7%)	616	3652	RdRp	ON236587 ^3^

^1^ HP: hypothetical protein; CP: coat protein; RdRP: RNA-dependent RNA polymerase; ^2^ complete genome sequence including 5′ and 3′ genome ends; ^3^ complete genome coding; DmFV1: Diplodia mutila fusagravirus 1; DsPV1: Diplodia seriata partitivirus 1; DsEV1: Diplodia seriata endornavirus 1; NpVV1: Neofusicoccum parvum victorivirus 1; NpNV3: Neofusicoccum parvum narnavirus 3; AtNsRV1: Alternaria tenuissima negative-strand RNA virus 1; DsMBV1: Diplodia seriata mycobunyavirus 1; DsNV1: Diplodia seriata narnavirus 1.

**Table 3:** Intraspecies nucleotide variability in the PCR fragment used for the detection of mycoviruses in the *Botryosphaeriaceae* collection.

Virus	Number of Positive Isolates	Range of Pairwise Nucleotide Divergence
AtNSRV1	3	1.5–10.2%
NpVV1	4	0–9.8%
NpMV3	3	0–4.2%
DsPV1	2	0.7–4.7%
NpEV1	6	0–11.5%
DsEV1	10	4.3–12.5%
NpNV3	2	0–2.6%
NlFV1	2	0
NlMV1	2	0

## Data Availability

The raw dsRNA-HTS data are freely available at www.ncbi.nlm.nih.gov/bioproject/PRJNA825664 (accessed on 12 April 2022).

## Supplementary Materials

The following supporting information can be downloaded at: https://www.mdpi.com/article/10.3390/v16030392/s1, Figure S1: Neighbor-joining tree reconstructed using the alignment of ORF1-deduced protein sequences from *Fusagraviridae* members; Figure S2: Neighbor-joining phylogenetic trees reconstructed using the nucleotide sequences of the PCR products generated for the detection of mycoviruses; Table S1: Summary of the viruses and primers included in the present study; Table S2: Percentage of amino acid identities in the proteins encoded by ORF1 and ORF2 of Diplodia mutila fusagravirus 1 isolate ARB45 with the corresponding proteins of *Fusagraviridae* members. References [44,45,47,54,56] are cited in the supplementary materials.
