# Supplementary material for: Characterization of the RNA Mycovirome Associated with Grapevine Fungal Pathogens: Analysis of Mycovirus Distribution and Their Genetic Variability within a Collection of Botryosphaeriaceae Isolates
Source: Viruses. 2024 Mar 1;16(3):392. doi: 10.3390/v16030392 (PMC10975779; doi:10.3390/v16030392)
Supplement: Supplementary file 1 [file viruses-16-00392-s001.zip › viruses-2787528-supplementary.pdf]

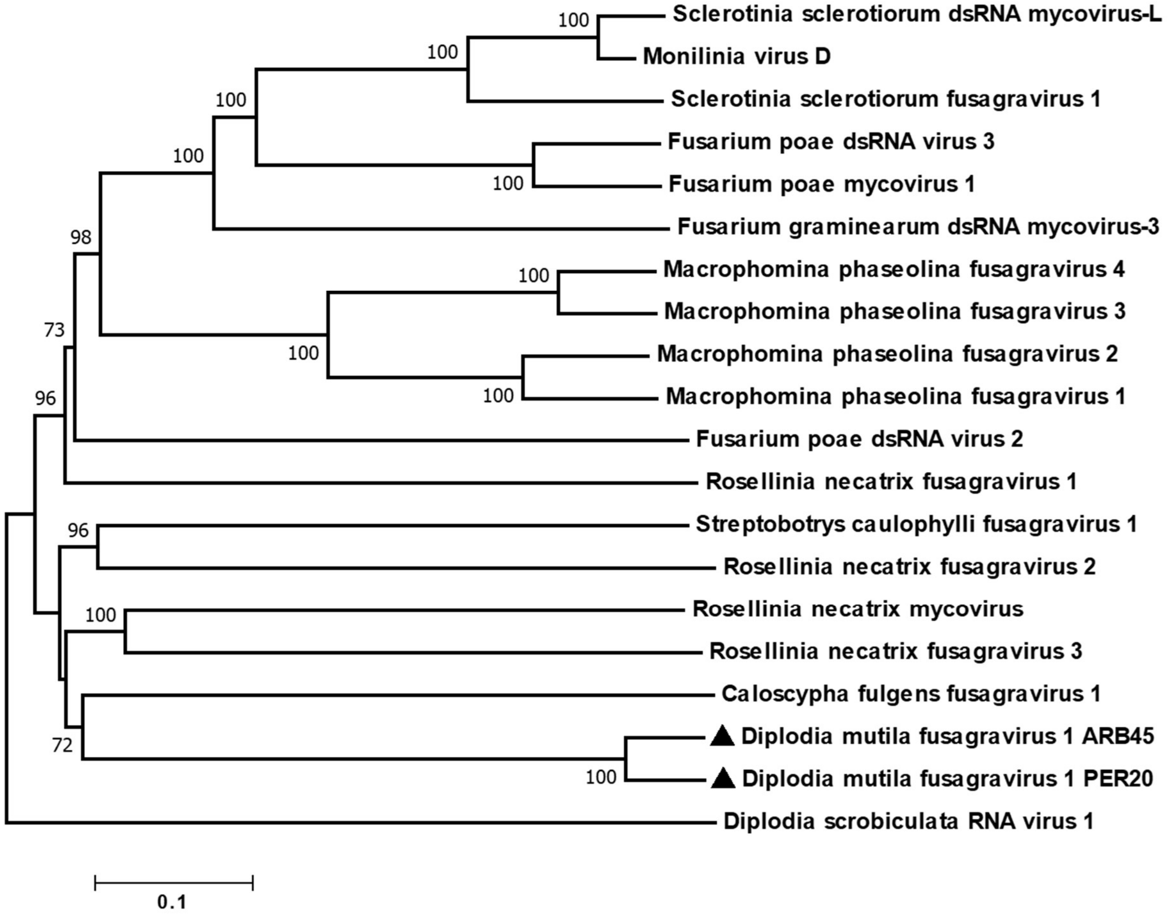

Figure S1. Neighbor-joining tree reconstructed using the alignment of ORF1-deduced protein sequences from Fusagraviridae members.

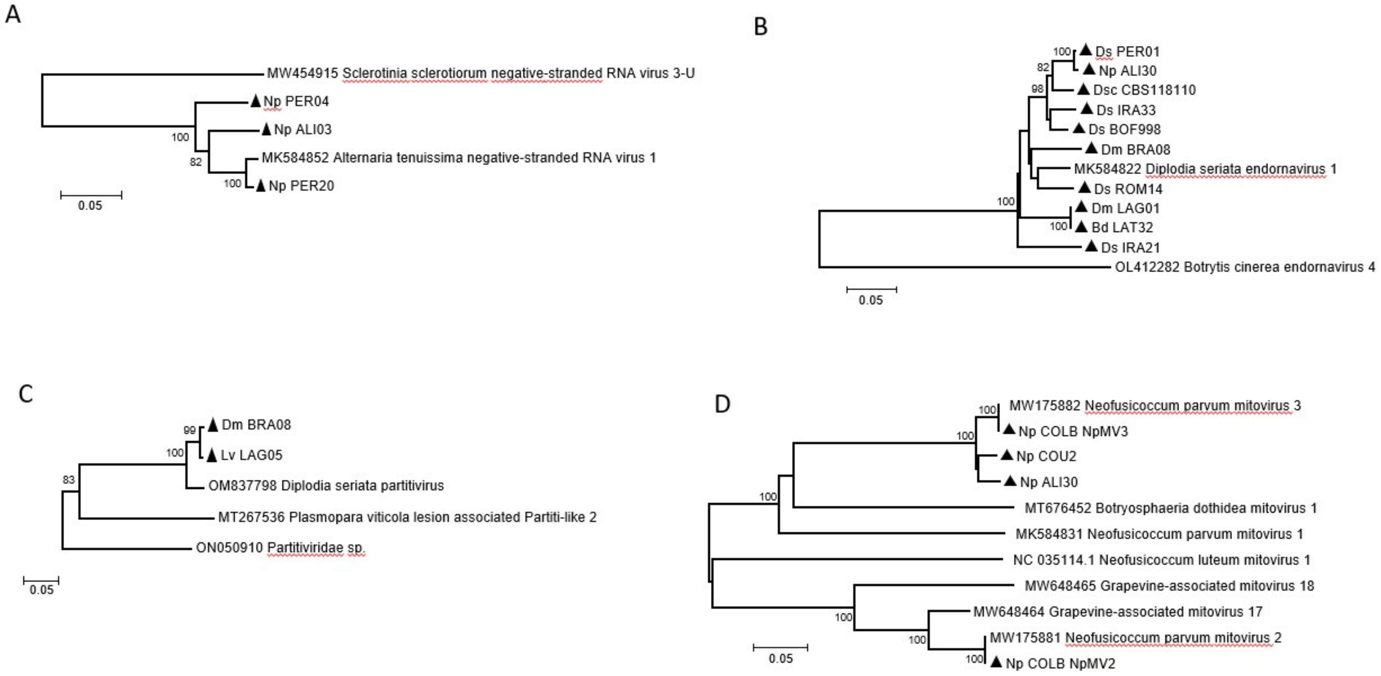

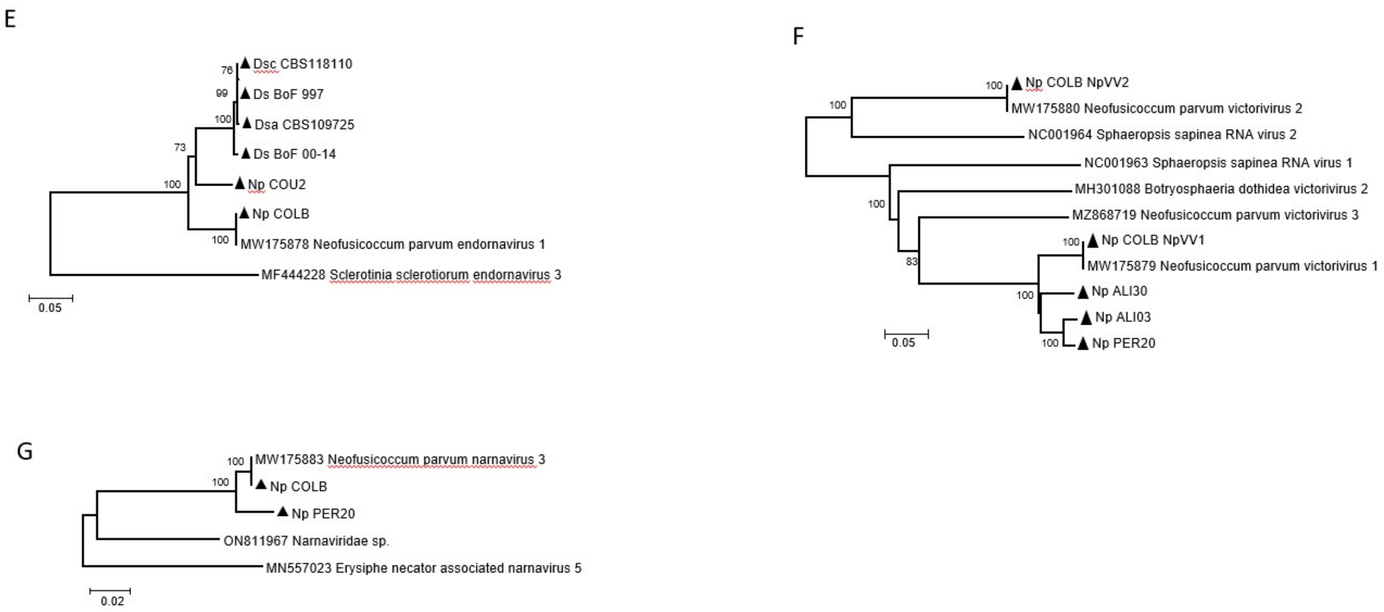

Figure S2. Neighbor-joining phylogenetic trees reconstructed using the nucleotide sequences of the PCR products generated for the detection of mycoviruses.

Table S1. Summary of the viruses and primers included in the present study.

Virus name	Viral family (type)	Primer	Sequence 5′-3′	Target	At	Accession number	References
Diplodia mutila fusagravirus 1 (DmFV1)	*Fusagraviridae* (dsRNA)	Fusa-F2	GCTACCCGCCGTCTGGCTATG	DmFV1 ORF1	58°C	ON236578 ON236579	This study
		Fusa-R2	CAGAGCGCGGAGCCGGTTCTG				
		ARB45-Div-5Race1	CGATAGCGACTTACCACCCGGAC-GACC	DmFV1 5′Race	68°C		
		ARB45-Div-5Race2	ACCTCGCTAACGGCGAGGGAT-TCTTCCG	DmFV1 5′Race	68°C		
		ARB45-Div-LD	CGACCTTCCCCCCGTGGCGGCGGA	DmFV1 3′Race	68°C		
Diplodia seriata mycobunyavirus 1 (DsBMV1)	*Bunyavirales* (-ssRNA)	DsBMV1-F1	GAACATGAGAGATTAAACAAGATG	DsBMV1 RdRp	57°C	ON236586	This study
		DsBMV1-R1	TCGTTCTTGTTCTGAATTCAG				
Diplodia seriata narnavirus 1 (DsNV1)	*Narnaviridae* (+ssRNA)	DsNV1-F1	GATCGGTAGATATGTTGGTATG	DsNV1 RdRp	54°C	ON236587	This study
		DsNV1-R1	GCTCATCATTATTGAACTCCAT				
Diplodia seriata endornavirus 1 (DsEV1)	*Betaendornaviridae* (+ssRNA)	BE-F1	AAGCGCATGGAAGACATGATG	DsEV1 RdRp	58°C	MK584822 ON236580 ON236581	[44], This study
		DsEV1-R	GACGCGATCTTCACTGAGGGA				
Alternaria tenuis-sima negative-stranded RNA vi-rus 1 (AtNRsV1)	*Mymonaviridae* (-ssRNA)	Mono-F1	TCTTGCTGCAATAGCTGAGATG	AtNSRV1 RdRp	58°C	MK584808 ON236577	[44], This study
		Mono R1	AGATTCGCCTCGGTGAGTACAA				
		PER20-AtNSRV1-5Race3	CAGAAACAGCATCGCGAAC-GACACTC	AtNSRV1 5′Race	68°C		
		PER20-AtNSRV1-LD4	CCACCGCGCAAACCAG-CATCTCGAC	AtNSRV1 3′Race	68°C		

Neofusicoccum parvum narnavirus 3 (NpNV3)	*Narnaviridae* (+ssRNA)	NpNV3-F1<br>NpNV3-R1	TACTGTTGATGGGCTGGTAATG<br>CCGGTACATACTGATCGGTGT	NpNV3 RdRp	58°C	MW175883<br>ON236576	[45], This study
		PER20-NpNV3-5Race	TCCCAAGAGAGGAGTTCTTCCCACATT	NpNV3 5′Race	64°C		
		PER20-NpNV3-LD	TCATCCTACACATGTGCCATTTAGTGG	NpNV3 3′Race	62°C		
Neofusicoccum parvum victorivirus 1 (NpVV1)	*Totiviridae* (dsRNA)	NpVV1-F1<br>NpVV1-R1	AGCCCTGATCCAGCTGCTCTC<br>GGTCACCATTTCGGTTGTGAG	NpVV1 RdRp	58°C	MW175879<br>ON236575	[45], This study
Diplodia seriata partitivirus 1 (DsPV1)	*Partitiviridae* (dsRNA)	Part-F1<br>Part-R1	TTGCATCAACGCTGTTCAAATG<br>GAGCAGCTGAATCGATAGGG	DsPV1 RdRp	58°C	OM837798<br>ON236582<br>ON236583<br>ON236584<br>ON236585	[47], This study
		BRA08-Part-Race2	TAATCGCCGTCATAACAACGCCTCGGTAG	DsPV1 5′Race	68°C		
Neofusicoccum parvum victorivirus 2 (NpVV2)	*Totiviridae* (dsRNA)	NpVV2-F1<br>NpVV2-R1	CGAGATGGTCTAGTCACCATG<br>GCTTCAGTTAAGCGCCCATAAG	NpVV2 RdRp	58°C	MW175880	[45]
Neofusicoccum parvum mitovirus 3 (NpMV3)	*Mitoviridae* (+ssRNA)	NpMV3-FD<br>NpMV3-RD	TATTCTTTCACTCCTTGGAGT<br>TCACATAATGAGACCATAACT	NpMV3 RdRp	52°C	MW175882	[45]
Neofusicoccum parvum mitovirus 2 (NpMV2)	*Mitoviridae* (+ssRNA)	NpMV2-F1<br>NpMV2-R1	ACCGATCGGTATCTCATCTTCT<br>CGGAAGCTACAGGAGACGAGAC	NpMV2 RdRp	58°C	MW175881	[45]
Neofusicoccum parvum endornavirus 1 (NpEV1)	*Alphaendornaviridae* (+ssRNA)	NpEV1-F1<br>NpEV1-R1	GGAACTTCATAGTGAACATGATG<br>GCCCATGGTTCTGGTTTCTG	NpEV1 RdRp	58°C	MW175878	[45]
Neofusicoccum parvum chrysovirus 1 (NpCV1)	*Chrysoviridae* (dsRNA)	NpCV1-F1<br>NpCV1-R1	GCACGCTGTCGTGCTGCCCATG<br>GATCGCGCTTACTCTTCGCCAT	NpCV1 RdRp	63°C	MK584816	[44]
Neofusicoccum parvum mitovirus 1 (NpMV1)	*Mitoviridae* (+ssRNA)	NpMV1-F1<br>NpMV1-R1	GGCTGCGAAAGCACCATACAT<br>CTGAATCCACCAGTTCGTTAT	NpMV1 RdRp	55°C	MK584831	[44]
Neofusicoccum parvum narnavirus 1 (NpNV1)	*Narnaviridae* (+ssRNA)	NpNV1-F1<br>NpNV1-R1	GCTGCGCTTCCAGTATAACATG<br>TTGGTCATCATATCTCCCATCAT	NpNV1 RdRp	55°C	MK584833	[44]

Neofusicoccum parvum narnavirus 2 (NpNV2)	*Narnaviridae* (+ssRNA)	NpNV2-F1	ATTGATCCATGCTTGCGCATGG	NpNV2 RdRp	59°C	MK584834	[44]
		NpNV2-R1	GAAAGTAACGAAACCCGATACAT				
Neofusicoccum parvum RNA virus 1 (NpVlV1)	Unclassified (+ssRNA)	NpVlV1-F1	ATGCCTGCTTGGCTTGCCAAG	NpVlV1 RdRp	53°C	MK584849	[44]
		NpVlV1-R1	TCCTCGTCACCGAATATCAT				
Neofusicoccum parvum ourmia-like virus 1 (NpOulV1)	*Botourmiaviridae* (+ssRNA)	NpOulV1-F1	ACGGGAACGCAACGCAGATG	NpOulV1 RdRp	59°C	MK584837	[44]
		NpOulV1-R1	GTACTGCTGTATCGTATCAACCAT				
Neofusicoccum luteum fusarivirus 1 (NlFV1)	*Fusariviridae* (+ssRNA)	NlFV1-F1	GATTTTGATGAATCCACTAAT	NlFV1 RdRp	50°C	NC_040791	[56]
		NlFV1-R1	CACTAAAGATGTTATTATTCAT				
Neofusicoccum luteum mitovirus 1 (NlMV1)	*Mitoviridae* (+ssRNA)	CAP037 Mito F1	CAAGATCAGTCAAGCCTACC	NlMV1	55°C	NC_035114	[54]
		CAP037 Mito R1	AGAAGGTTATCATGTATAGGC				

Table S2. Percentage of amino acid identities in the proteins encoded by ORF1 and ORF2 of Diplodia mutila fusagravirus 1 isolate ARB45 with the corresponding proteins of *Fusagraviridae* members.

Virus	ORF1	ORF2
Diplodia mutila fusagravirus 1 PER20	90.1%	92.1%
Diplodia scrobiculata RNA virus 1	9.9%	69.9%
Streptobotrys caulophylli fusagravirus 1	19.1%	32.4%
Rosellinia necatrix fusagravirus 2	15.7%	31%
Caloscypha fulgens fusagravirus 1	20.3%	30.5%
Fusarium graminearum dsRNA mycovirus 3	19.3%	30.5%
Fusarium poae dsRNA virus 3	21.3%	30.2%
Fusarium poae mycovirus 1	21.2%	30.5%
Sclerotinia sclerotiorum fusagravirus 1	19.7%	29.4%
Rosellinia necatrix fusagravirus 3	18.1%	29%
Rosellinia necatrix mycovirus	20%	28.9%
Rosellinia necatrix fusagravirus 1	17.4%	25.6%
